# Supplementary material for: Internal climate variability and projected future regional steric and dynamic sea level rise
Source: Nat Commun. 2018 Mar 14;9:1068. doi: 10.1038/s41467-018-03474-8 (PMC5852151; doi:10.1038/s41467-018-03474-8)
Supplement: Supplementary file 1 — Supplementary Information [file 41467_2018_3474_MOESM1_ESM.pdf]

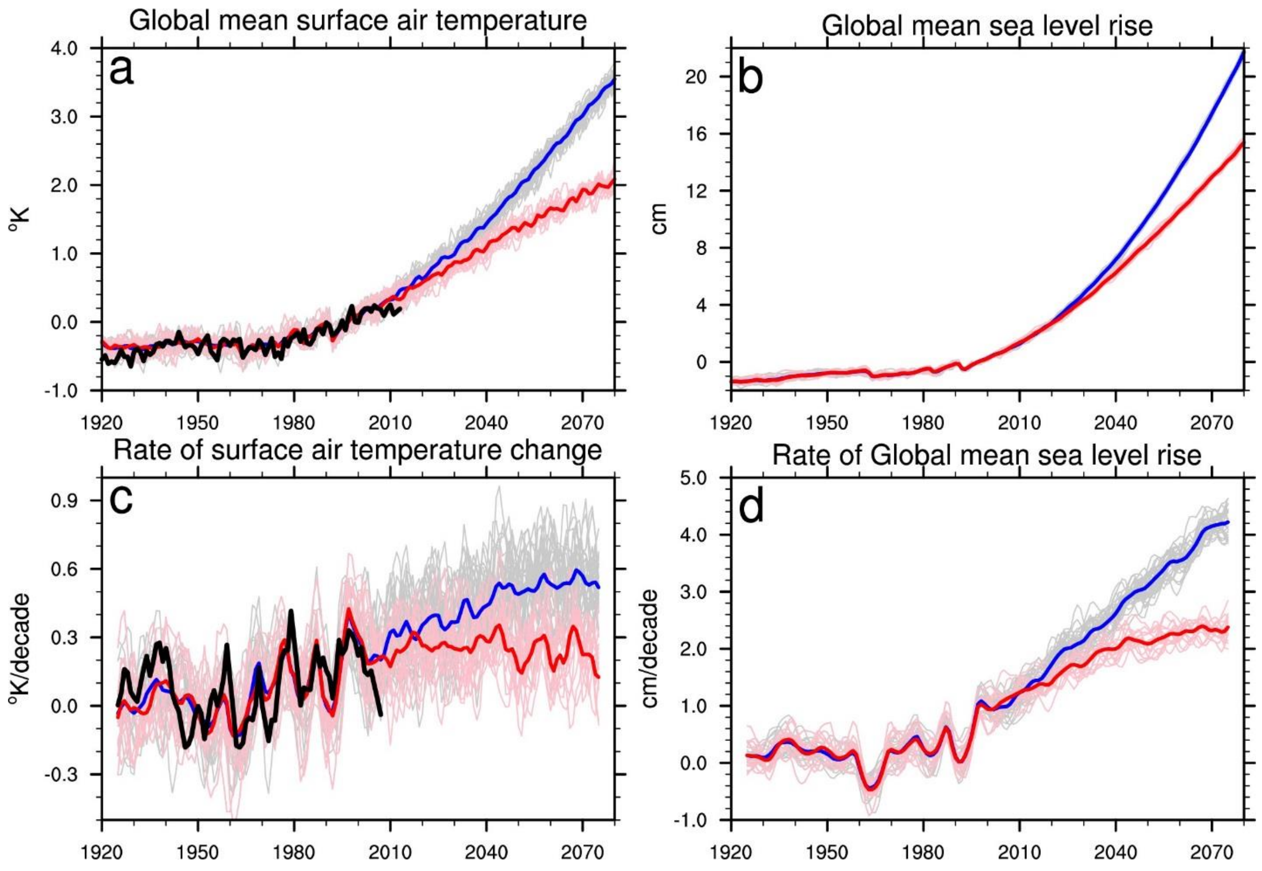

Supplementary Figure 1. Global mean surface air temperature and sea level anomalies from 1920 to 2080 relative to the mean of 1986-2005 and the running decadal trends. Panel **a** is the global mean surface temperature, panel **b** is the global mean steric sea level rise, panel **c** is the running decadal trend of the global mean temperature, and panel **d** is the running decadal trend of the global mean steric sea level rise. The black solid line is for observations, the red line is the ensemble mean for RCP4.5 and the blue line is the ensemble mean for RCP8.5. The gray and pink lines represent individual ensemble members.

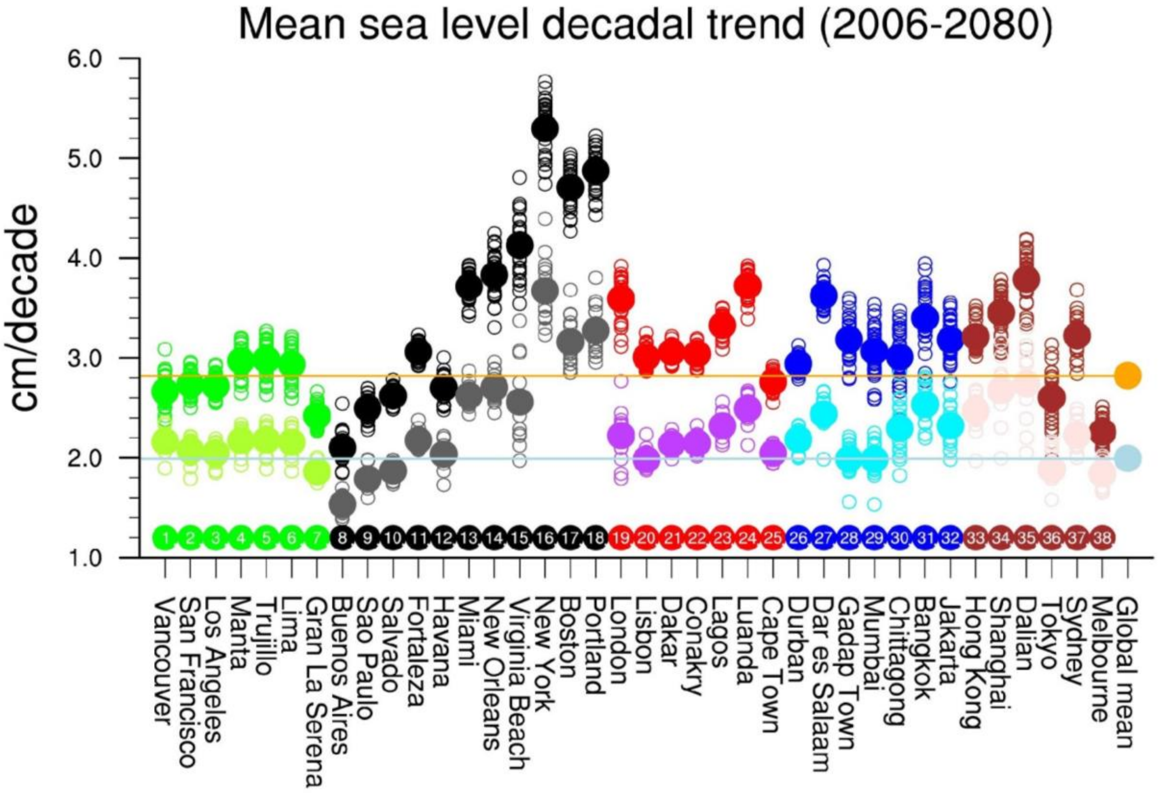

Supplementary Figure 2. Sea level decadal trend for the global mean and selected coastal cities from 2006-2080. The color codes are green (yellow green) represents east Pacific cities for RCP8.5 (RCP4.5); black (gray) west Atlantic cities; red (purple) east Atlantic cities; blue (light blue) Indian Ocean cities; brown (light pink) west Pacific cities; dark orange (light gray) the global mean.

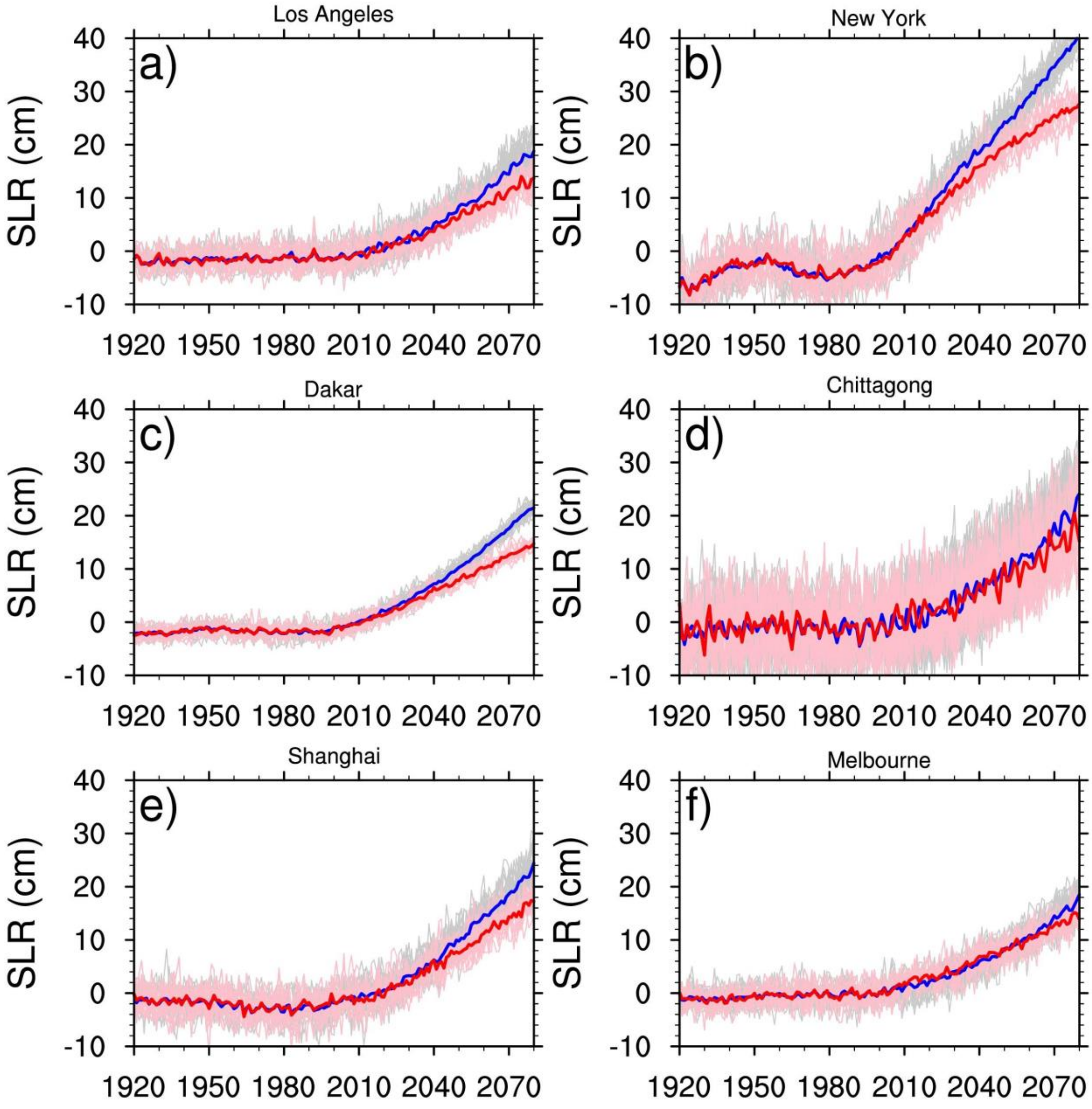

Supplementary Figure 3. Time evolving sea level rise for 6 selected coastal cities. Data is from 1920 to 2080 under 20th century all forcings from 1920 to 2005 and under RCP 8.5 (blue/gray lines) and RCP4.5 (red/pink lines) scenarios after 2005. The blue/red lines are the ensemble mean for RCP8.5 and RCP4.5 (20th century), respectively

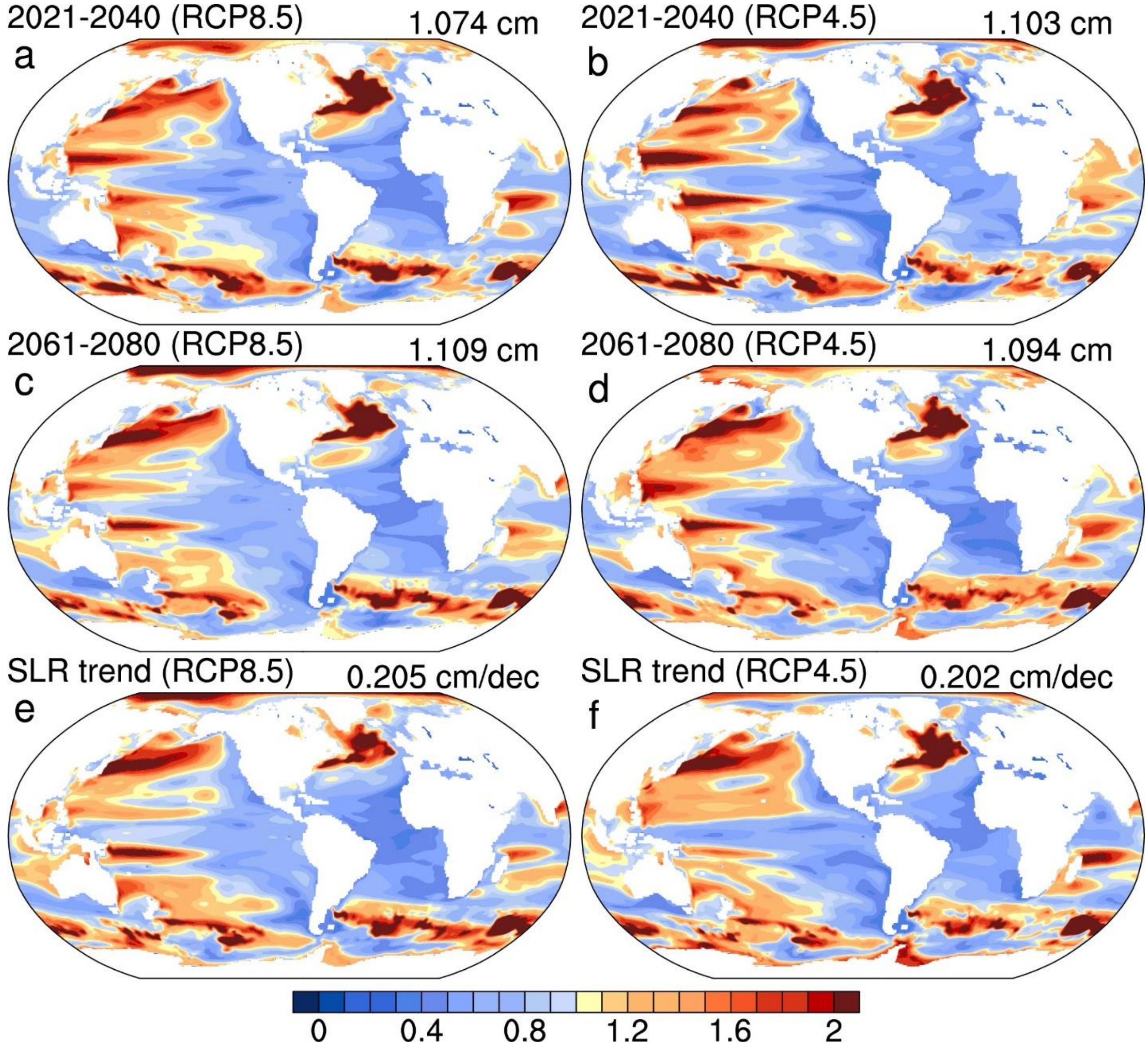

Supplementary Figure 4. Ratios of the intra-ensemble standard deviation relative to the global mean. The numbers on top left of each panel are the ensemble global mean value, and shading in all panels are weighted by those global mean values. Panels a and b are averaged over 2021-2040 and c and d averaged over 2061-2080. Panels e and f is the intra-ensemble standard deviation of the decadal trend over the period 2006-2080. The contour interval is 0.1.

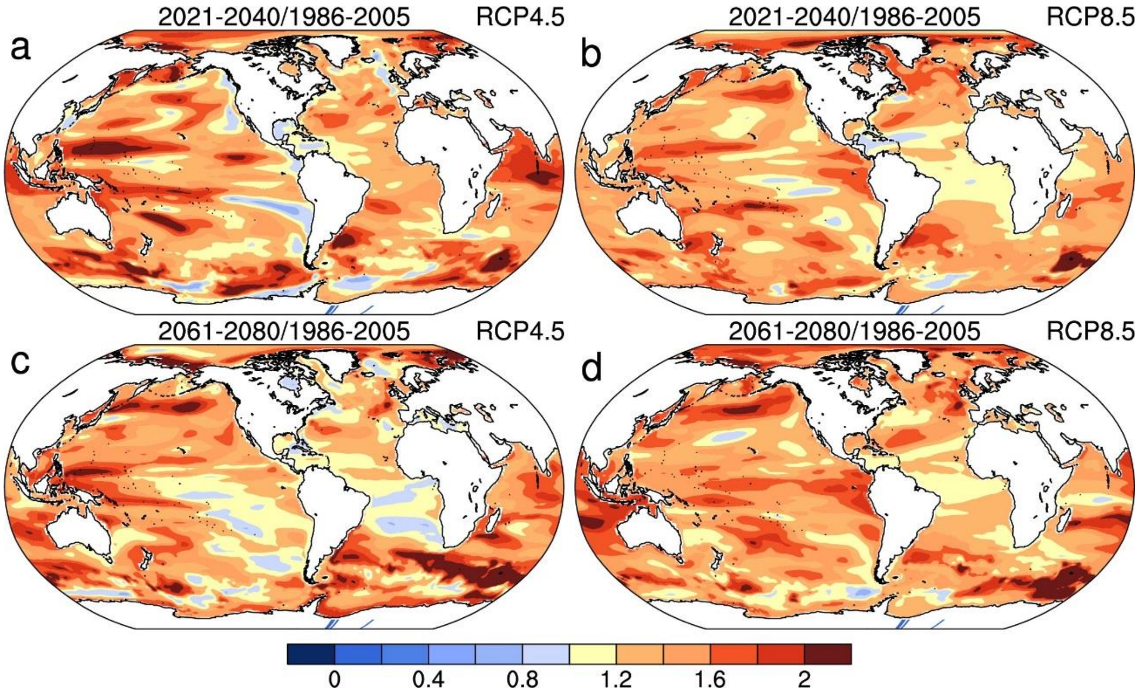

Supplementary Figure 5. Ratios of the decadal mean SLR standard deviation between RCP4.5 (RCP8.5) and 20th century. The decadal mean for the 20th century is over 1986-2005, and the decadal mean for the 21st century is 2021-2040 and 2061-2080. The intra-ensemble SLR variance is calculated as the variance across each individual ensembles. Ratios greater (less) than one represent a larger (smaller) across ensemble variance. Panels **a** and **c** are for RCP4.5, and panels **b** and **d** are for RCP8.5. This figure represents the changes of the SLR variance over time.

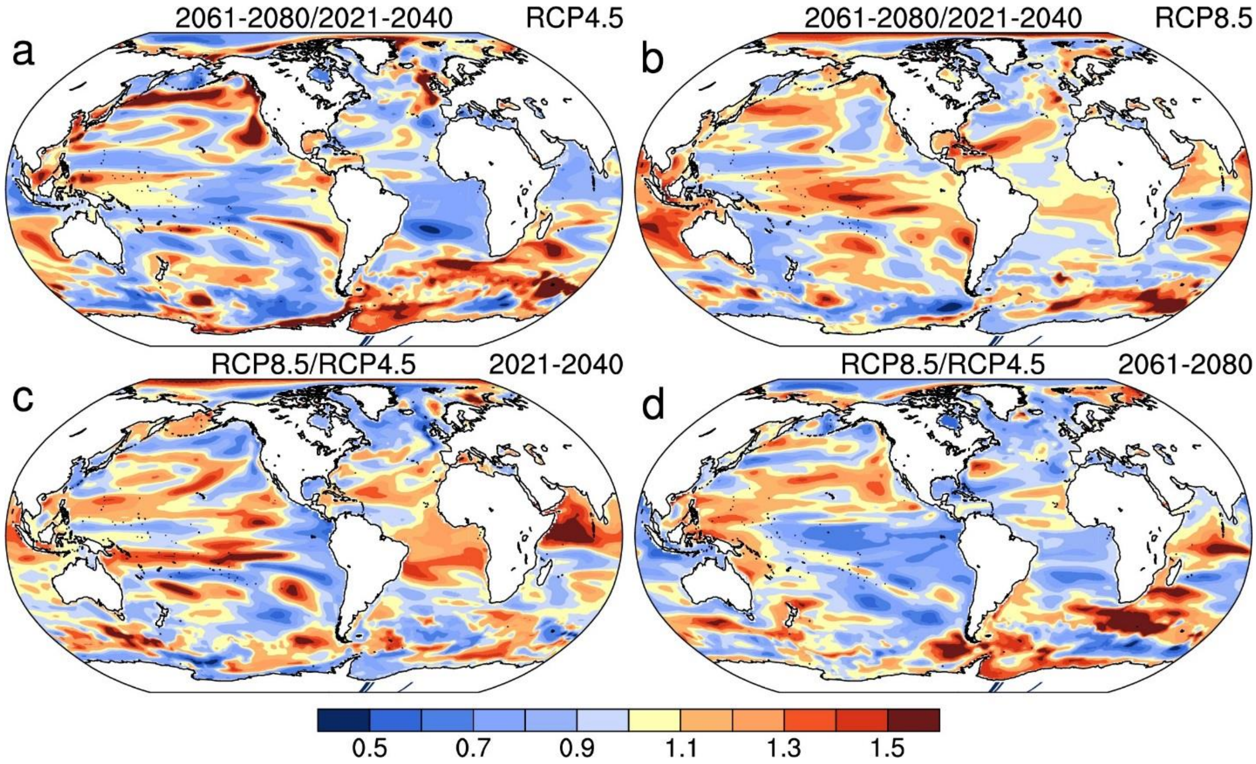

Supplementary Figure 6. Ratios of the decadal mean SLR standard deviation. The decadal mean used here is 2021-2040 and 2061-2080. Ratios greater (less) than one represent a larger (smaller) across ensemble variance. Panels **a** and **b** represent the decadal mean SLR variance changes between 2021-2040 and 2061-2080 for RCP4.5 and RCP8.5, respectively. Panels **c** and **d** are the ratios of the SLR standard deviation between RCP8.5 and RCP4.5 for period 2021-2040 (**c**) and 2061-2080 (**d**). Ratios greater (less) than one represent a larger (smaller) across ensemble variance.

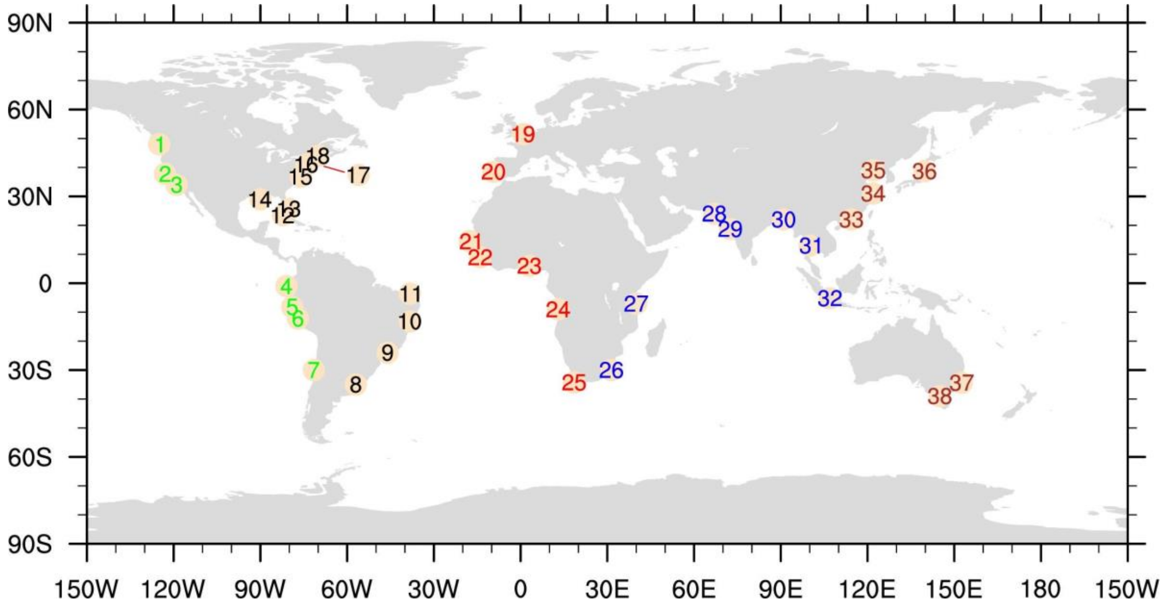

Supplementary Figure 7. Geographic map to show the physical locations of selected coastal cities. Color code: green represents east Pacific cities; black west Atlantic cities; red east Atlantic cities; blue Indian Ocean cities; brown west Pacific cities; dark orange the global mean. The numbers in the plot correspond to cities: 1 Vancouver; 2 San Francisco; 3 Los Angeles; 4 Manta; 5 Trujillo; 6 Lima; 7 Gran La Serena; 8 Buenos Aires; 9 Sao Paulo; 10 Salvado; 11 Fortaleza; 12 Havana; 13 Miami; 14 New Orleans; 15 Virginia Beach; 16 New York; 17 Boston; 18 Portland; 19 London; 20 Lisbon; 21 Dakar; 22 Conakry; 23 Lagos; 24 Luanda; 25 Cape Town; 26 Durban; 27 Dar es Salaam; 28 Gadap Town; 29 Mumbai; 30 Chittagong; 31 Bangkok; 32 Jakarta; 33 Hong Kong; 34 Shanghai; 35 Dalian; 36 Tokyo; 37 Sydney; 38 Melbourne.

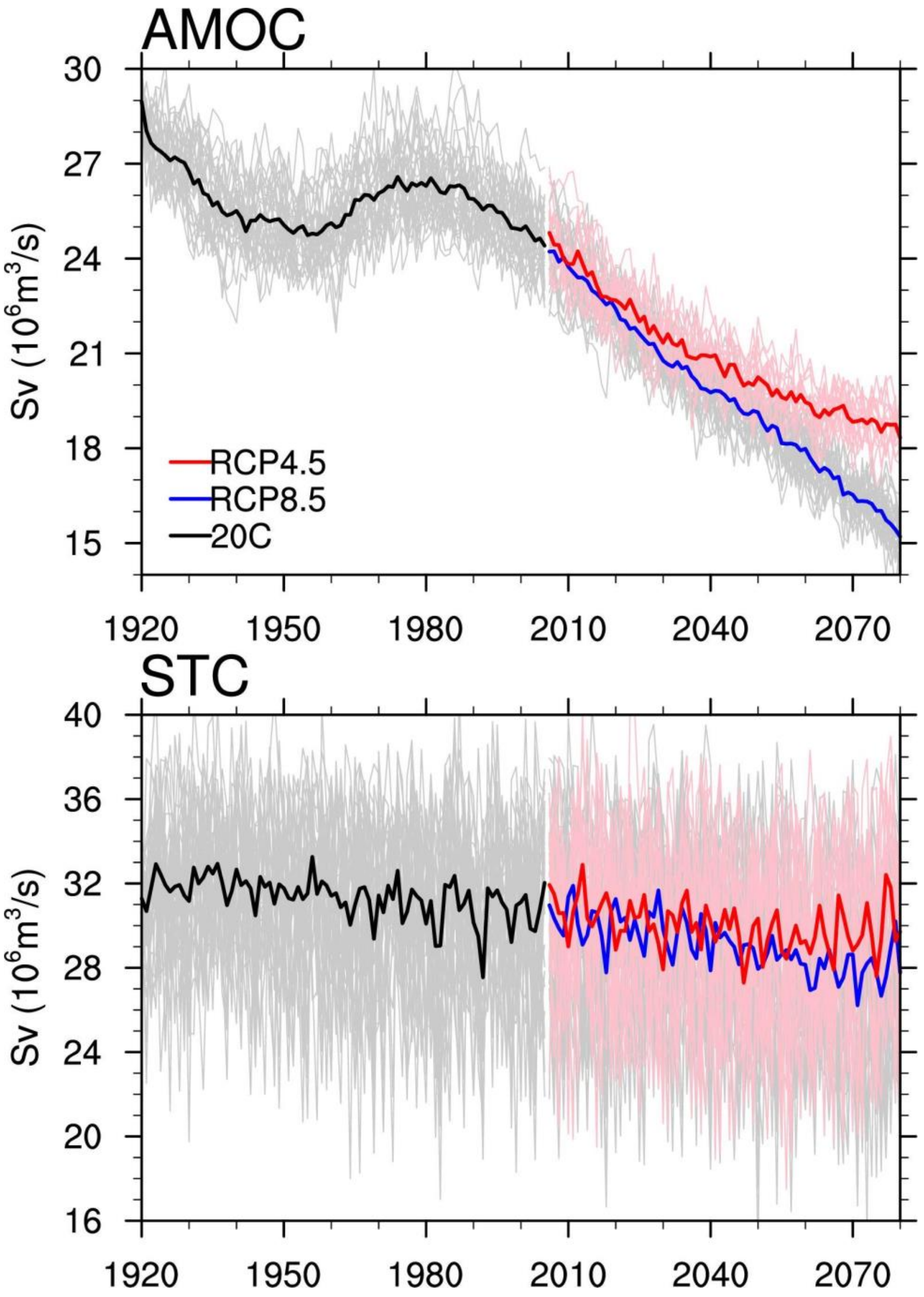

Supplementary Figure 8. The time evolving Atlantic meridional overturning circulation (AMOC, top panel) and the Pacific subtropical cell (STC, bottom panel) index from 1920 to 2080. Black/Blue/red lines are the ensemble mean for 20th century (1920-2005), RCP8.5 and RCP4.5 (2006-2080), and gray/pink lines for individual members. The AMOC index is defined as the maximum of the Atlantic meridional streamfunction below 500 meter depth. The STC index is defined as the difference between the mean Pacific meridional streamfunction of 0-5ºN in upper 150 meter.

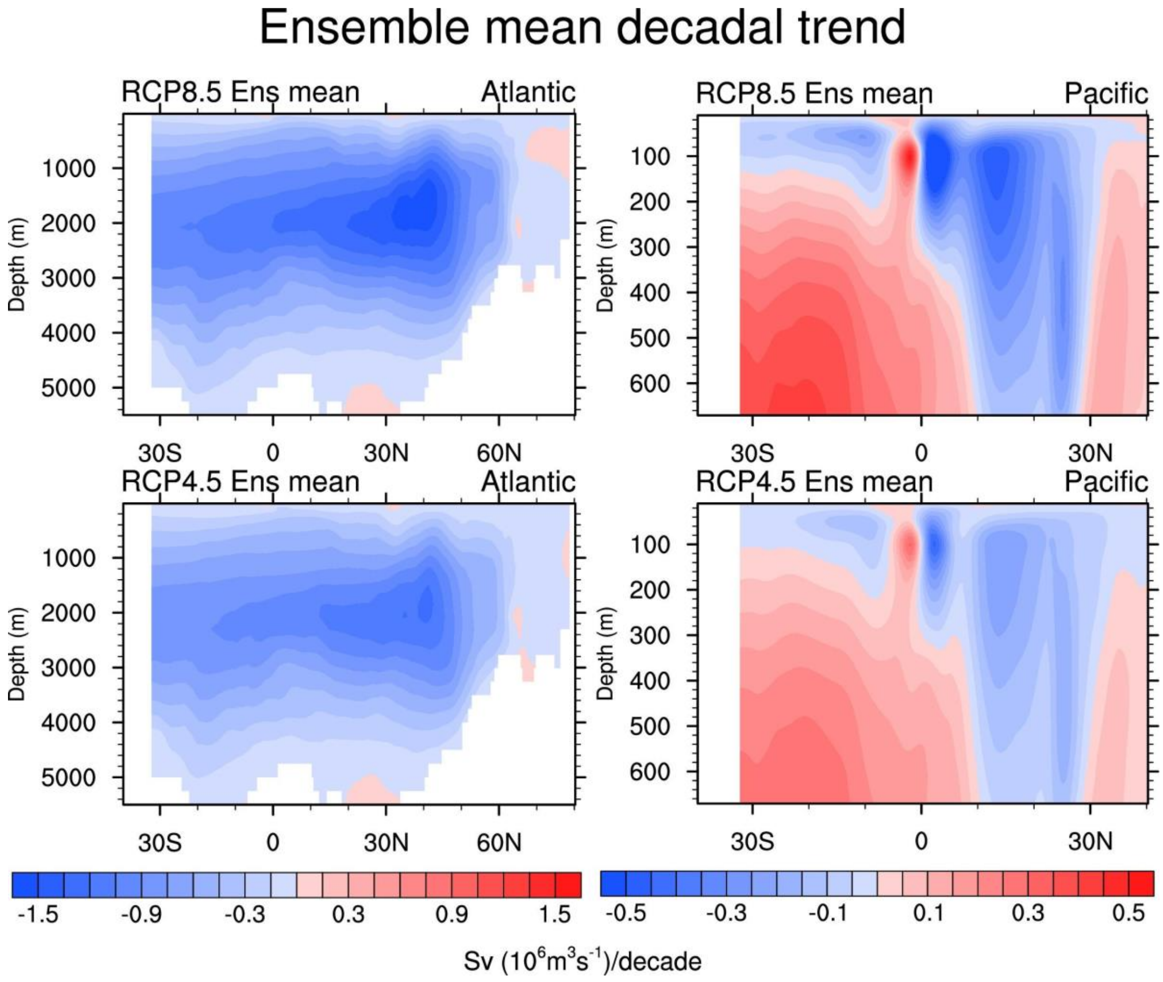

Supplementary Figure 9. Decadal trend of the meridional streamfunction in Atlantic and Pacific from 2006 to 2080. Left panels are for the Atlantic meridional streamfunction trend representing the trend of the Atlantic meridional overturning circulation (AMOC), and the right panels are the Pacific meridional streamfunction trend representing the trend of the Pacific subtropical cells (Pacific STCs). The contour interval for Atlantic meridional streamfunction is 0.15 Sv/decade and 0.05 Sv/decade for Pacific.

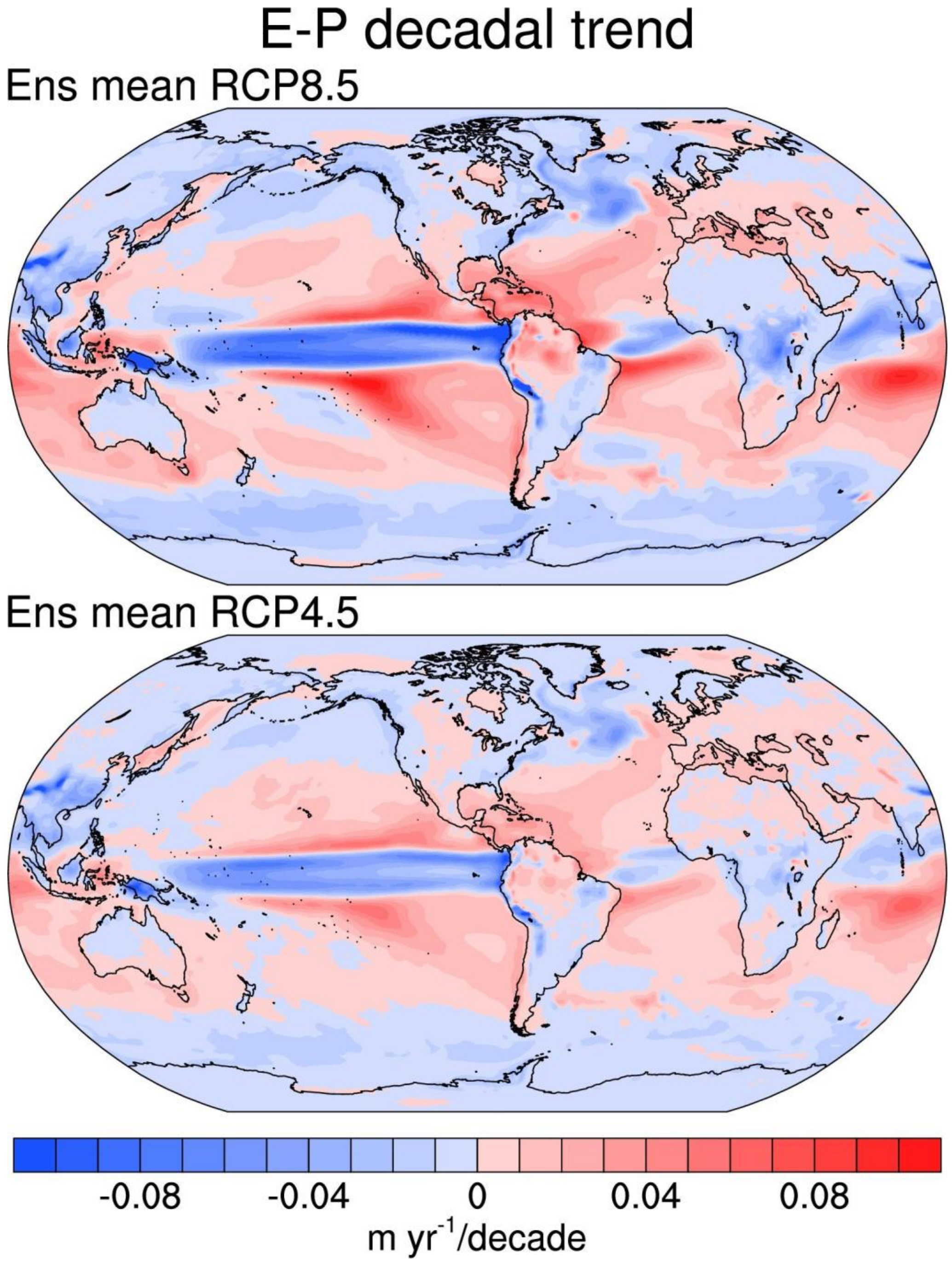

Supplementary Figure 10. Ensemble mean decadal trend of evaporation minus precipitation for RCP8.5 and RCP4.5. The contour interval is 0.01m yr^{-1}/decade.

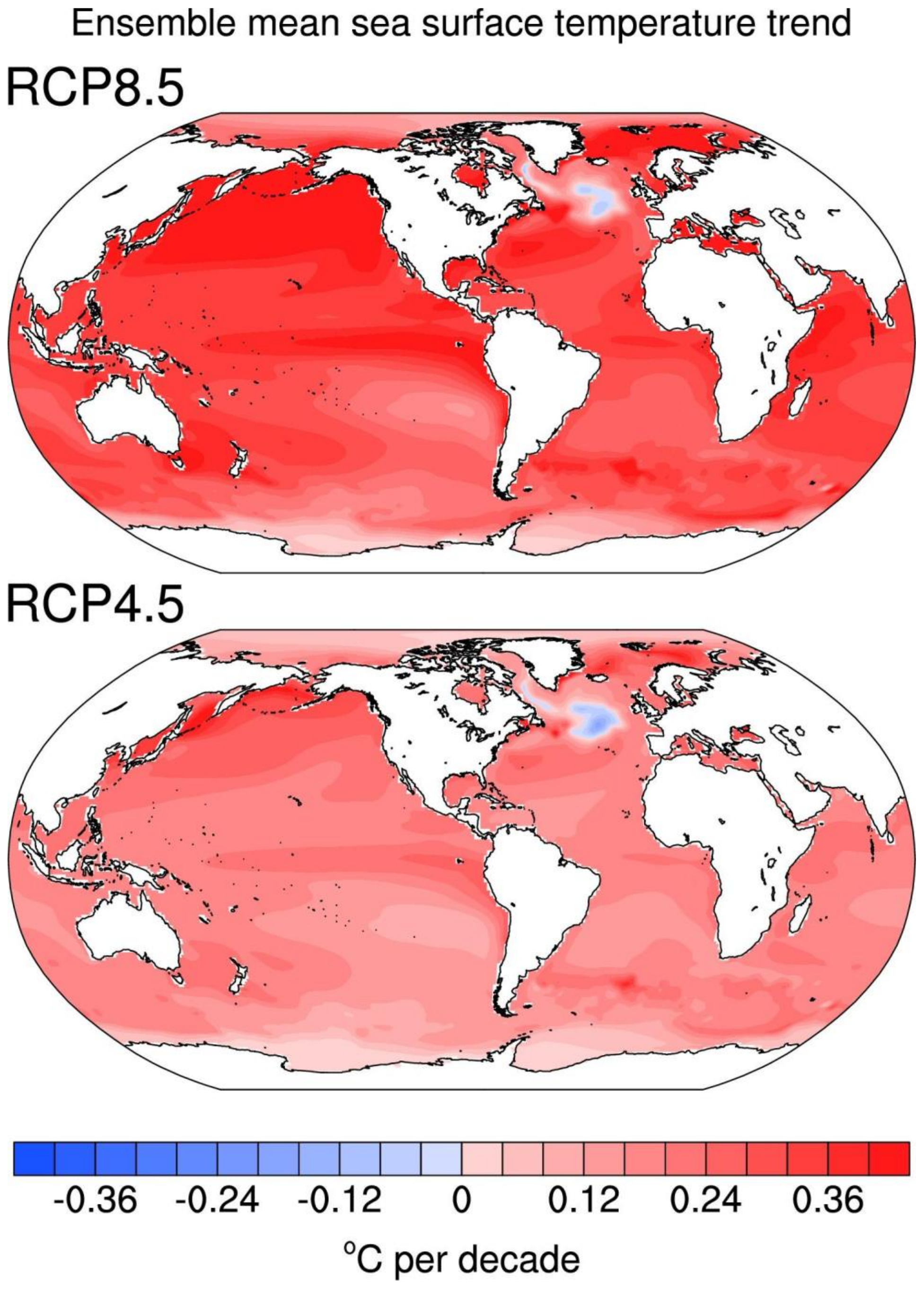

Supplementary Figure 11. Ensemble mean sea surface temperature decadal trend for RCP8.5 and RCP4.5 ensembles from 2006 to 2080. The contour interval is 0.06°C.

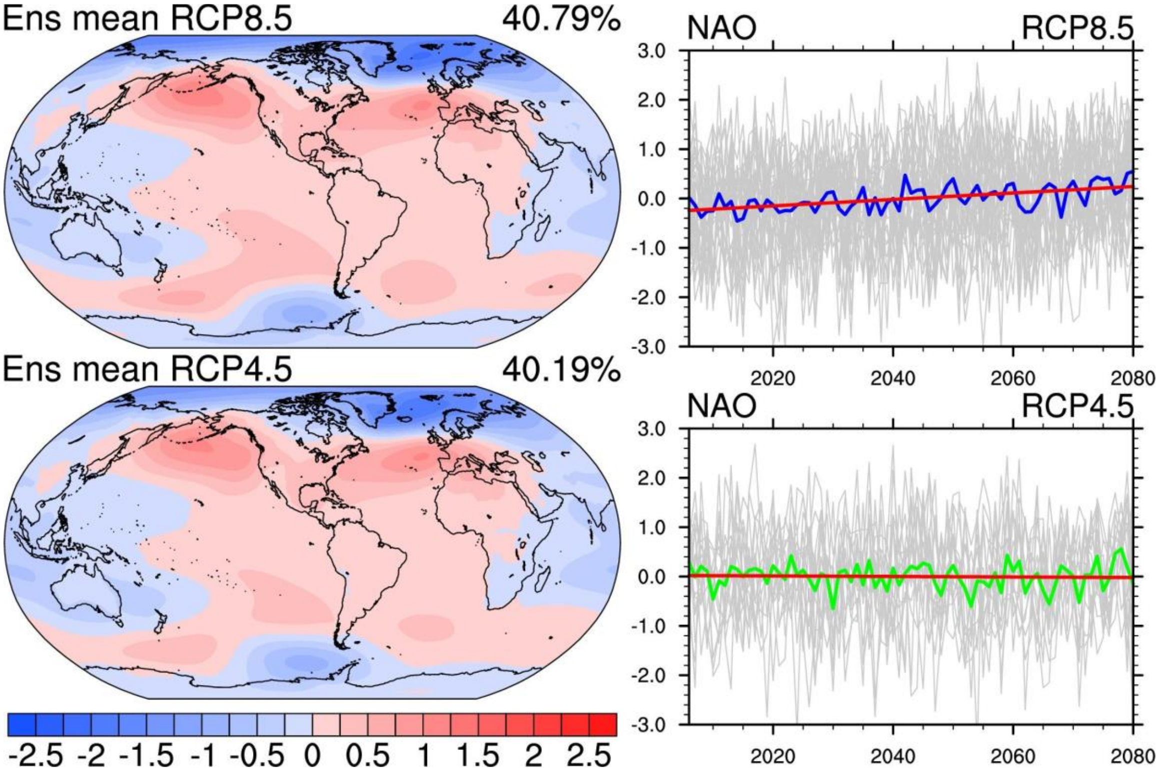

Supplementary Figure 12. Regression of the NAO index on sea level pressure and the NAO index. Left panels are the ensemble mean regression pattern of the annual mean NAO index and sea level pressure for RCP8.5 (top left) and RCP4.5 (bottom left). The percentage number is the sea level pressure variance explained by the NAO. Right panels are the NAO index for RCP8.5 (top right) and RCP4.5 (bottom right). The gray lines in the right panels are the annual mean NAO index for each individual member, the blue/green line is the ensemble mean NAO index for RCP8.5/RCP4.5, and the red lines are the linear fit of the NAO index.

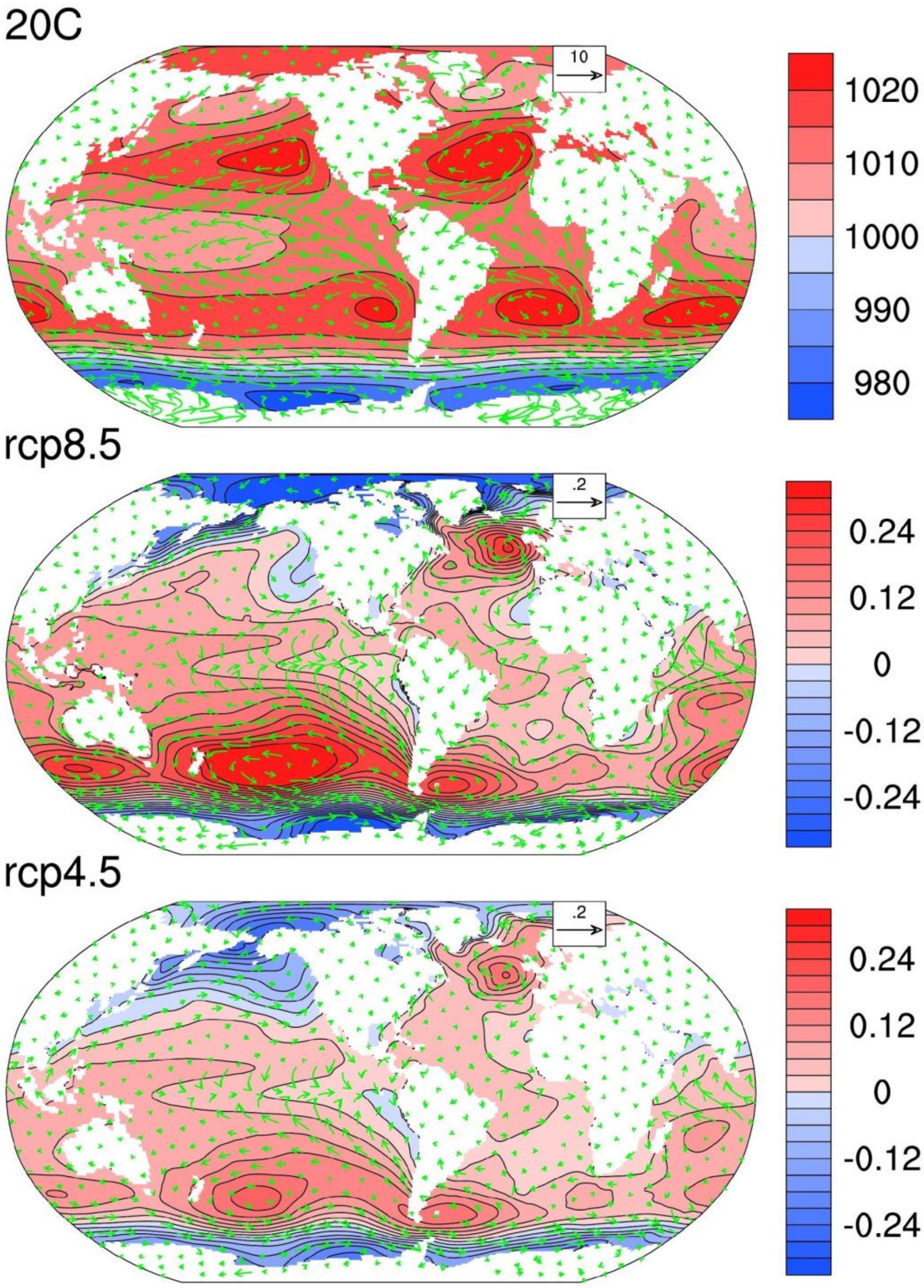

Supplementary Figure 13. Sea level pressure and surface wind. The top panel is the ensemble mean sea level pressure (hPa, shading and contour) and surface wind (m/s, vectors) for the late 20th century (20C, averaged over 1986-2005). The mid and bottom panels are the ensemble mean decadal trend of the sea level pressure (hPa/decade, shading and contour) and wind (m/s/decade, vectors) from 2006-2080 for RCP8.5 and RCP4.5.

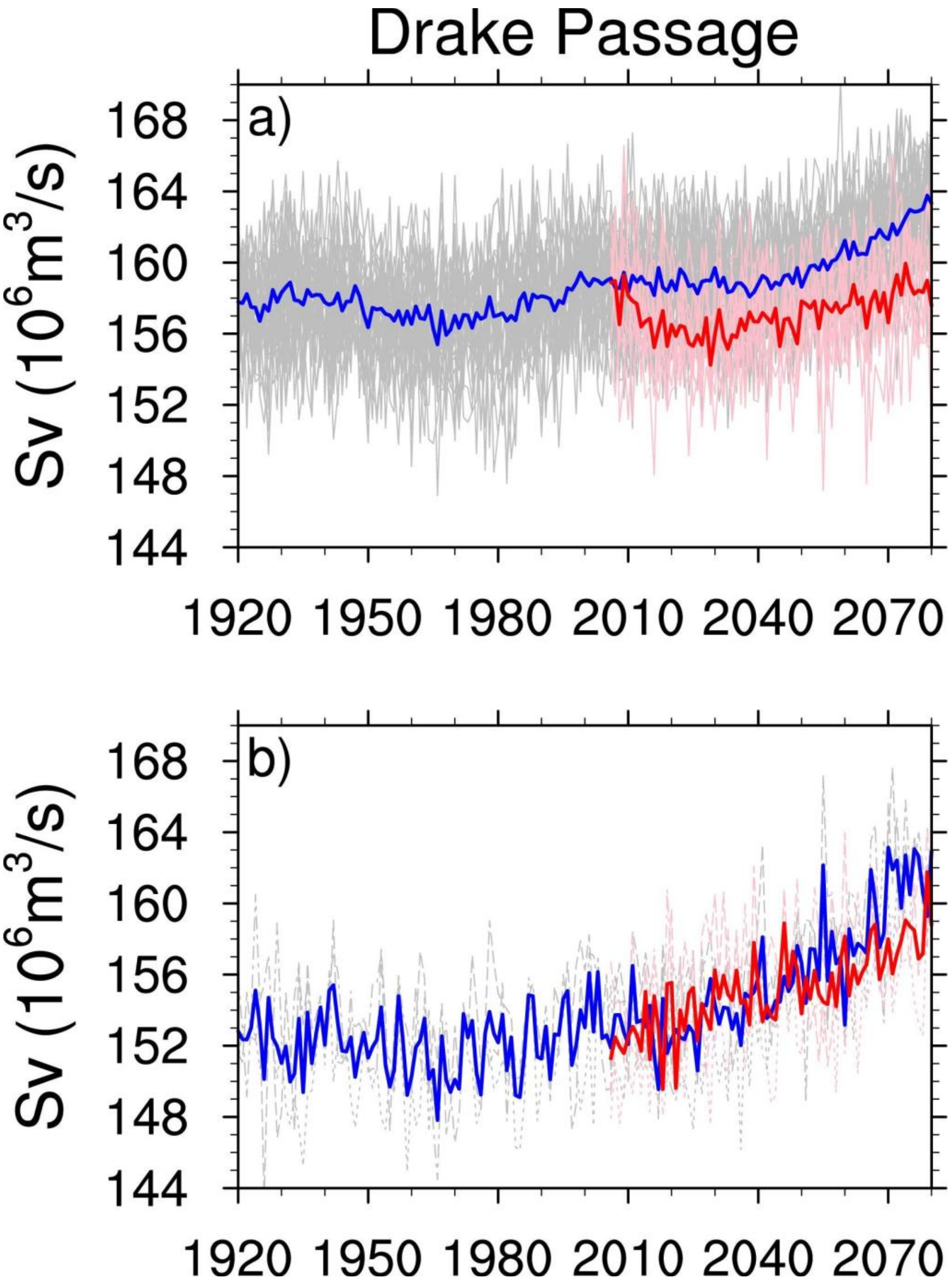

Supplementary Figure 14. Time evolving Drake Passage transport from 1920 to 2080 for the large ensemble simulations (a) and the CESM1 coupled model intercomparison phase 5 (CMIP5) simulations (b). Blue is the ensemble mean for the 20th century all forcing simulation (1920 to 2005) and for RCP8.5 (2006-2080), and red is the ensemble mean for RCP4.5. The gray (20th century and RCP8.5) and pink (RCP4.5) are for individual ensemble members.

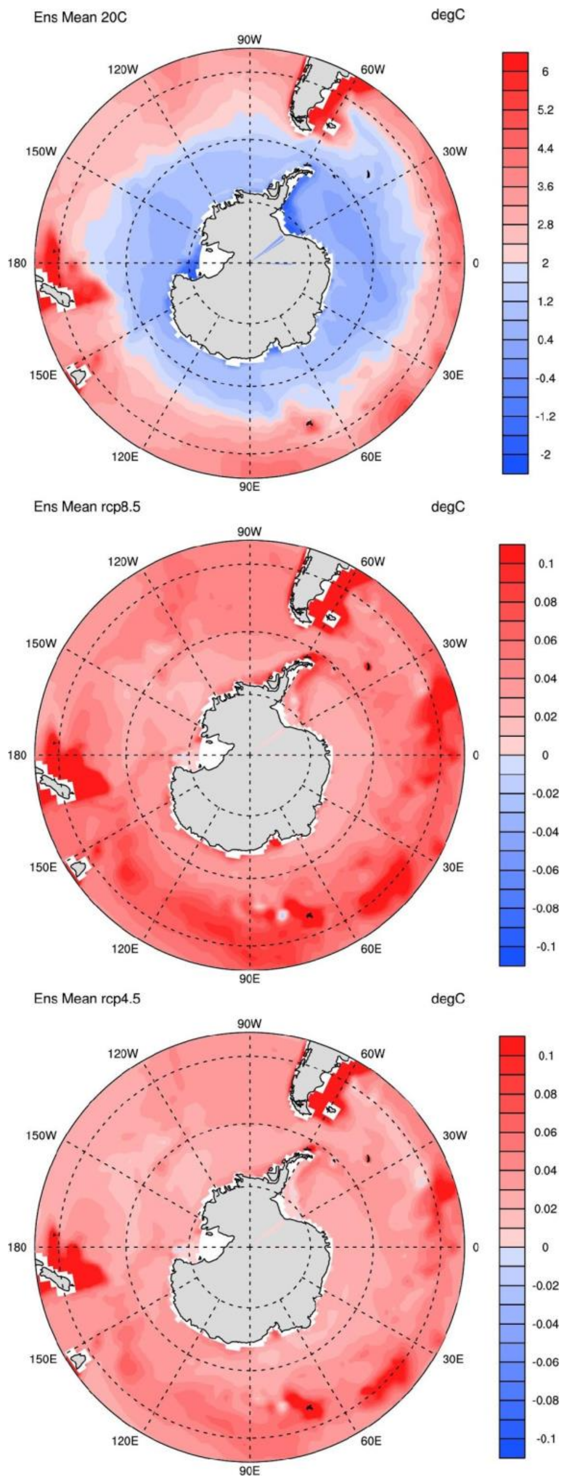

Supplementary Figure 15. Vertically integrated mean ocean temperature. Top panel is the mean ocean temperature averaged over all ensemble members between 1986 and 2005; mid and bottom panels are the decadal trend of the ocean temperature for RCP8.5 and RCP4.5. The contour interval for top panel is 0.4^{o}C and for mid and bottom panels is 0.01^{o}C/decade.

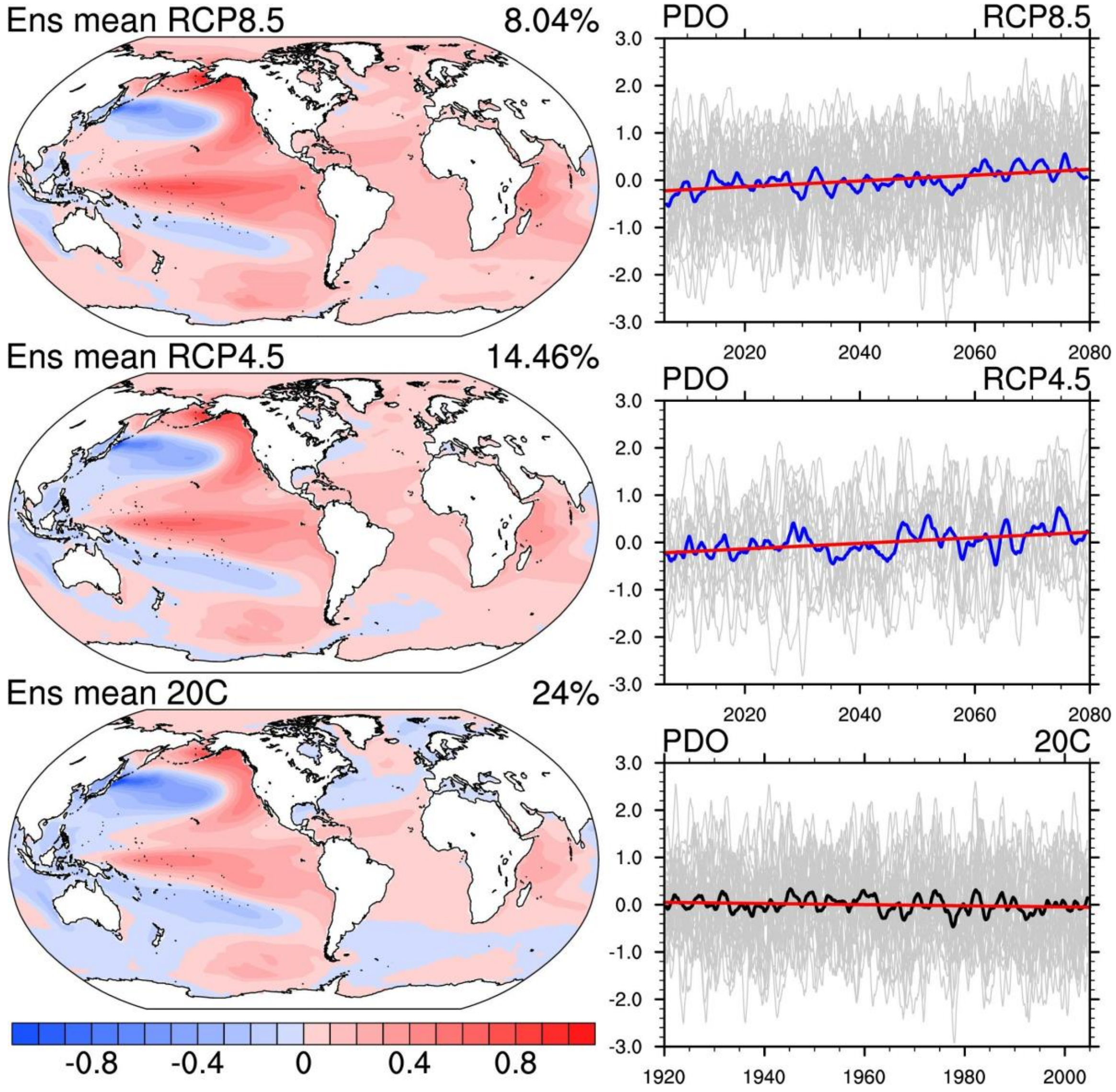

Supplementary Figure 16. Left panels are the regression of the Pacific decadal oscillation (PDO) onto the ensemble mean sea surface temperature, and right panels are the PDO time series. The percentage number is the SST variance explained by the PDO. The gray lines in the right panels are the annual mean PDO index for each individual member, the blue/black line is the ensemble mean PDO index for RCP8.5, RCP4.5 and 20th century, and the red lines are the linear fit of the PDO index. Top and mid panels are for the RCP8.5 and RCP4.5, respectively, and the bottom panel is for the 20th century simulations.
