# Supplementary material for: Internal climate variability and projected future regional steric and dynamic sea level rise
Source: Nat Commun. 2018 Mar 14;9:1068. doi: 10.1038/s41467-018-03474-8 (PMC5852151; doi:10.1038/s41467-018-03474-8)
Supplement: Supplementary file 3 — Description of Additional Supplementary Files [file 41467_2018_3474_MOESM3_ESM.docx]

**Description of Additional Supplementary Files**

File Name: Supplementary Data 1

Description: Global and regional sea level rise in 21st century (cm)

File Name: Supplementary Data 1

Description: Global and regional sea level rise decadal trend (cm/decade)
