# Supplementary material for: Internal climate variability and projected future regional steric and dynamic sea level rise
Source: Nat Commun. 2018 Mar 14;9:1068. doi: 10.1038/s41467-018-03474-8 (PMC5852151; doi:10.1038/s41467-018-03474-8)
Supplement: Supplementary file 4 — Supplementary Data 1 [file 41467_2018_3474_MOESM4_ESM.docx]

Supplementary Data 1 Global and regional sea level rise in 21^st^ century (cm)

|  | Averaged over 2061-2080 relative to the mean of 1986-2005 | | | | | | | |
| --- | --- | --- | --- | --- | --- | --- | --- | --- |
|  | RCP8.5 | | | | RCP4.5 | | | |
| Cities | Mean | Perc | Max | Min | Mean | Perc | Max | Min |
| Global Mean  Vancouver  San Francisco  Los Angeles  Manta  Trujillo  Lima  Gran La Serena  Buenos Aires  Sao Paulo  Salvado  Fortaleza  Havana  Miami  New Orleans  Virginia Beach  New York  Boston  Portland  London  Lisbon  Dakar  Conakry  Lagos  Luanda  Cape Town  Durban  Dar es Salaam  Gadap Town  Mumbai  Chittagong  Bangkok  Jakarta  Hong Kong  Shanghai  Dalian  Tokyo  Sydney  Melbourne | 17.78±0.15  14.10±0.70  15.24±0.55  15.25±0.49  16.69±0.65  16.95±0.70  16.68±0.69  14.37±0.42  13.54±0.43  17.26±0.43  17.16±0.29  19.16±0.27  15.65±0.48  22.41±0.49  22.97±0.84  28.44±0.90  35.03±0.79  30.62±0.69  31.65±0.70  23.42±0.97  18.29±0.40  17.98±0.34  17.93±0.33  21.50±0.34  25.00±0.49  16.37±0.29  17.42±0.32  21.29±0.74  22.51±0.79  21.30±0.82  17.25±1.08  17.53±1.11  17.94±0.76  16.72±0.56  18.84±1.01  21.77±1.35  15.51±1.00  21.86±0.60  14.12±0.62 | 100  79  86  86  94  95  94  81  76  97  97  108  88  126  129  160  197  172  178  132  103  101  101  121  141  92  98  120  127  120  97  99  101  94  106  122  87  123  79 | 18.09  15.89  16.88  16.67  18.25  18.58  18.28  15.63  15.05  17.98  17.75  19.70  16.79  23.38  24.75  30.68  36.88  32.03  33.03  25.14  19.13  18.67  18.67  22.14  25.82  17.13  18.10  23.26  24.01  23.16  20.06  20.20  19.91  17.87  20.73  24.67  17.24  23.17  15.09 | 17.44  12.69  14.21  14.37  15.60  15.83  15.64  13.72  12.92  16.34  16.67  18.70  14.85  21.62  21.38  26.67  33.64  29.31  30.34  20.54  17.42  17.36  17.27  20.94  23.99  15.74  16.47  20.13  21.57  20.12  14.83  15.58  16.52  15.33  16.60  18.64  13.49  20.28  12.79 | 13.16±0.16  11.05±0.59  11.35±0.48  11.27±0.51  11.89±0.45  12.19±0.54  12.15±0.56  11.25±0.32  11.25±0.53  13.93±0.49  13.44±0.39  14.19±0.24  11.97±0.41  15.95±0.36  16.25±0.66  19.12±1.15  25.18±0.96  21.34±0.65  22.03±0.66  15.14±1.02  12.08±0.36  12.56±0.40  12.72±0.36  15.80±0.32  18.09±0.52  12.64±0.43  13.67±0.52  14.53±0.86  15.89±0.66  15.47±0.78  14.23±1.08  13.76±1.01  14.05±0.69  13.06±0.76  14.31±0.85  15.65±1.24  11.53±0.87  16.96±0.54  12.60±0.35 | 100  84  86  86  90  93  92  85  85  102  102  108  91  121  123  145  191  162  167  115  92  95  97  119  137  96  104  110  121  118  108  105  107  99  109  119  88  129  96 | 13.43  12.01  12.16  11.85  12.66  13.15  13.06  11.69  12.09  15.11  14.21  14.60  12.49  16.59  17.33  20.89  26.60  22.52  23.22  17.24  12.64  13.62  13.65  16.54  19.17  13.17  14.52  16.03  17.09  16.72  16.09  15.12  14.89  14.54  15.73  17.63  12.94  17.48  13.03 | 12.84  10.06  10.45  10.57  10.98  10.95  10.95  10.84  10.15  12.92  12.68  13.68  11.04  15.32  15.08  16.14  23.31  20.23  20.93  13.55  11.32  11.85  12.06  15.23  17.16  11.94  12.48  13.09  14.77  14.10  11.71  11.91  12.66  12.05  13.04  13.57  10.23  15.50  11.77 |

Red indicates that the local SLR is below the global mean. In the table, “Mean” is the ensemble mean, “Perc” is the percent of the SLR in selected cities relative to the global mean, “Max/Min” is the maximum/minimum among all ensemble members.
