# Supplementary material for: Internal climate variability and projected future regional steric and dynamic sea level rise
Source: Nat Commun. 2018 Mar 14;9:1068. doi: 10.1038/s41467-018-03474-8 (PMC5852151; doi:10.1038/s41467-018-03474-8)
Supplement: Supplementary file 5 — Supplementary Data 2 [file 41467_2018_3474_MOESM5_ESM.docx]

Supplementary Data 2 Global and regional sea level rise decadal trend (cm/decade)

|  | 2006-2080 | | | | | | | |
| --- | --- | --- | --- | --- | --- | --- | --- | --- |
|  | RCP8.5 | | | | RCP4.5 | | | |
| Cities | Mean | Perc | Max | Min | Mean | Perc | Max | Min |
| Global Mean  Vancouver  San Francisco  Los Angeles  Manta  Trujillo  Lima  Gran La Serena  Buenos Aires  Sao Paulo  Salvado  Fortaleza  Havana  Miami  New Orleans  Virginia Beach  New York  Boston  Portland  London  Lisbon  Dakar  Conakry  Lagos  Luanda  Cape Town  Durban  Dar es Salaam  Gadap Town  Mumbai  Chittagong  Bangkok  Jakarta  Hong Kong  Shanghai  Dalian  Tokyo  Sydney  Melbourne | 2.82±.03  2.66±.16  2.74±.11  2.72±.10  2.97±.16  2.98±.17  2.94±.16  2.42±.10  2.10±.14  2.50±.11  2.62±.08  3.06±.08  2.70±.12  3.71±.14  3.83±.22  4.13±.30  5.30±.27  4.71±.19  4.88±.20  3.59±.20  3.01±.10  3.06±.08  3.04±.09  3.33±.10  3.72±.13  2.76±.09  2.94±.07  3.62±.12  3.18±.22  3.07±.25  3.01±.27  3.40±.28  3.18±.21  3.21±.14  3.45±.22  3.79±.28  2.60±.26  3.22±.18  2.26±.13 | 100  94  97  96  105  106  104  86  74  89  93  109  96  132  136  146  188  167  173  127  107  109  108  118  132  98  104  128  113  109  107  121  113  114  122  134  92  114  80 | 2.89  3.09  2.96  2.94  3.20  3.27  3.21  2.66  2.54  2.70  2.78  3.23  3.01  3.93  4.25  4.81  5.77  5.04  5.22  3.92  3.25  3.22  3.19  3.50  3.92  2.92  3.13  3.93  3.60  3.54  3.47  3.95  3.55  3.52  3.79  4.19  3.13  3.68  2.51 | 2.76  2.38  2.50  2.55  2.65  2.63  2.60  2.27  1.85  2.28  2.49  2.92  2.48  3.42  3.31  3.54  4.74  4.27  4.43  3.11  2.86  2.92  2.88  3.09  3.39  2.55  2.79  3.42  2.79  2.58  2.38  2.77  2.77  3.01  3.05  3.08  1.98  2.84  2.08 | 1.99±.03  2.16±.11  2.07±.12  2.05±.10  2.17±.12  2.18±.13  2.16±.13  1.86±.07  1.54±.09  1.79±.10  1.87±.08  2.17±.09  2.04±.12  2.62±.11  2.70±.14  2.56±.38  3.67±.32  3.16±.24  3.27±.24  2.22±.24  1.98±.09  2.14±.08  2.15±.09  2.32±.11  2.49±.14  2.05±.07  2.18±.10  2.44±.17  1.99±.17  1.98±.19  2.30±.28  2.53±.19  2.32±.21  2.47±.19  2.68±.27  2.74±.34  1.88±.24  2.23±.14  1.84±.12 | 100  109  104  103  109  110  109  93  77  90  94  109  103  132  136  129  184  159  164  112  99  108  108  117  125  103  110  123  100  99  116  127  117  124  135  138  94  112  92 | 2.04  2.35  2.22  2.19  2.33  2.37  2.35  2.00  1.70  1.98  1.98  2.38  2.22  2.87  2.96  3.37  4.39  3.68  3.80  2.77  2.24  2.28  2.33  2.56  2.67  2.15  2.33  2.67  2.21  2.25  2.84  2.81  2.74  2.68  3.16  3.30  2.17  2.49  2.02 | 1.93  1.90  1.79  1.85  1.92  1.89  1.87  1.75  1.39  1.60  1.73  2.08  1.73  2.44  2.45  1.97  3.23  2.85  2.96  1.79  1.88  1.99  2.02  2.13  2.13  1.94  2.00  1.99  1.56  1.53  1.82  2.20  1.98  1.97  2.00  2.05  1.27  2.00  1.66 |

Red indicates that the local SLR decadal trend is below the global mean decadal trend. The “Mean”, “Perc”,“Max”, and “Min” represent the same as in Table 1.
